# Developmental Origins of Metaflammation; A Bridge to the Future Between the DOHaD Theory and Evolutionary Biology

**DOI:** 10.3389/fendo.2022.839436

**Published:** 2022-02-03

**Authors:** Hiroaki Itoh, Megumi Ueda, Misako Suzuki, Yukiko Kohmura-Kobayashi

**Affiliations:** Department of Obstetrics and Gynecology, Hamamatsu University School of Medicine, Hamamatsu, Japan

**Keywords:** developmental origins of health and disease (DOHaD), metabolic syndrome, obesity, pregnancy, adipose tissue

## Abstract

Metabolic syndrome refers to obesity-associated metabolic disorders that increase the risk of type 2 diabetes, coronary diseases, stroke, and other disabilities. Environmental imbalance during the early developmental period affects health and increases susceptibility to non-communicable diseases, including metabolic syndrome, in later life; therefore, the Developmental Origins of Health and Disease (DOHaD) theory was established. According to the DOHaD theory, the hypothesis of the energy-saving ‘Thrifty Phenotype’ in undernourished fetuses is one of the well-accepted schemes as a risk of developing metabolic syndrome. This phenotype is evolutionarily advantageous for survival of the fittest in a hangry environment after birth, a strong selection pressure, but increases the risk of developing metabolic syndrome under an obesogenic diet according to the ‘Mismatch’ hypothesis. Increasing evidences support that chronic inflammation pathophysiologically connects obesity to metabolic disorders in metabolic syndrome, leading to the concept of ‘Metaflammation’. ‘Metaflammation’ in humans is proposed to originate from the evolutionary conservation of crosstalk between immune and metabolic pathways; however, few studies have investigated the contribution of evolutionary maladaptation to the pathophysiology of ‘Metaflammation’. Therefore, it is promising to investigate ‘Metaflammation’ from the viewpoint of selective advantages and its ‘Mismatch’ to an unexpected environment in contemporary lifestyles, in consideration of the principal concept of evolutionarily conserved nutrient sensing and immune signaling systems.

## Introduction

Metabolic syndrome refers to the co-occurrence of cardiovascular risk factors, including obesity-associated metabolic disorders, such as insulin resistance, atherogenic dyslipidemia, and hypertension, and is now a global public health issue despite being initially reported in Western countries (1). The prevalence of metabolic syndrome was recently reported to be higher in the urban populations of some developing countries than in Western countries, which has, in turn, increased the prevalence of type 2 diabetes, coronary diseases, stroke, and other disabilities (2).

Numerous epidemiological and animal studies demonstrated that environmental disturbances in the early critical period have an impact on health and increase susceptibility to non-communicable diseases, such as metabolic syndrome, in later life; therefore, the theory of Developmental Origins of Health and Disease (DOHaD) was established (3–6). According to the DOHaD theory, one of the well-accepted proposals for the risk of developing metabolic syndrome is the hypothesis of the energy-saving ‘Thrifty Phenotype’ in undernourished fetuses, which is evolutionarily advantageous for survival of the fittest in a starved environment after birth, a strong selection pressure, but increases the risk of metabolic syndrome under an obesogenic diet according to the ‘Mismatch’ hypothesis (7). This concept connects metabolic syndrome to the maladaptation of the evolutionarily acquired plasticity of metabolic regulation against the selection pressure of starvation.

Recent studies reported that chronic inflammation is pathophysiologically associated with obesity and metabolic disorders in metabolic syndrome; therefore, the concept of “Metaflammation” has been established (8, 9). ‘Metaflammation’ in humans is proposed to originate from the evolutionary conservation of crosstalk between immune and metabolic pathways, for example, based on the composition of the fat body of *Drosophila melanogaster* (9). In the evolutionary history of humans, immune and metabolic crosstalk appeared to be associated, at least partly, with responses and/or adaptation to the selection pressures of infection and/or starvation; however, to the best of our knowledge, few studies have focused on its contribution to the pathophysiology of ‘Metaflammation’ in metabolic syndrome. On the other hand, according to the DOHaD hypothesis, offspring with the energy-saving ‘Thrifty Phenotype’ (10) are predisposed to metabolic syndrome under an obesogenic diet according to the ‘Mismatch’ hypothesis (7).

In this mini review, we introduce the relationship between the ‘Thrifty Phenotype’ and metabolic syndrome in the DOHaD scheme as well as that between ‘Metaflammation’ and evolutionarily conserved nutrient sensing and immune signaling systems. We also discuss the importance of investigating ‘Metaflammation’ from the viewpoint of selective advantages and its ‘Mismatch’ to unexpected modern environments in consideration of the DOHaD concept, in addition to the principal concept of evolutionarily conserved nutrient sensing and immune signaling systems.

## The ‘Thrifty Phenotype’ Hypothesis in the DOHaD Theory; Starvation as a Selection Pressure

Initial evidence to support the concept of DOHaD was the deterioration of health in adulthood of British small babies with low birth weight (11, 12) and Dutch fetuses with undernourishment due to maternal starvation in World War II (13, 14). Rapid infantile growth, presumably indicative of an abundant nutrient supply after birth, particularly with low birth weight, is also causatively associated with obesity and/or metabolic syndrome in later life (15–19). These findings suggest that the continuous trajectory from an undernourished environment during the fetal period to an excessive nutrient supply after birth specifically leads to metabolic disruptions in later life. Hales and Barker proposed the ‘Thrifty Phenotype’ hypothesis, in which the body size of fetuses is reduced as an adaption to an insufficient energy supply *in utero* through the acquisition of the permanent energy-saving phenotype, resulting in a low birth weight (10, 20, 21). The ‘Thrifty Phenotype’ in offspring is hypothesized to be advantageous for survival of the fittest in a starved environment because of low energy demands, but increases the risk of diabetes and/or obesity under an obesogenic diet (10, 21) due to reduced insulin sensitivity, a predisposition to authentic and ectopic fat accumulation, and a lower respiratory oxygen quotient, which are risk factors for metabolic syndrome (20–23).

Starvation is one of strongest selection pressures from an evolutionary viewpoint not only in humans, but also in animals (24). The ‘Thrifty Phenotype’ is acquired phenotypic plasticity that changes offspring into energy-saving individuals in response to the presence or absence of a starved environment after birth as an adaptation to the selection pressure of repeated starvation waves (25). Based on the long evolutionary history of humans with repeating periods of starvation, the recent era of an overwhelming food supply in developed and some rapidly developing countries is an evolutionary exception. Therefore, it is plausible that the developmentally acquired plasticity of the ‘Thrifty Phenotype’ with energy-saving metabolic regulation mismatches an environment with an excess energy supply, thereby increasing the risk of obesity and diabetes, the so-called state of metabolic syndrome.

Gluckman and Hanson proposed not only the ‘Mismatch’ hypothesis (7), but also the ‘Predictive Adaptive Responses (PARs)’ hypothesis (26, 27). In the intrauterine setting, PARs primarily function to improve future fitness to expected conditions after birth, such as starvation, through evolutionarily acquired phenotypic plasticity for adaptation (26). The ‘Mismatch’ hypothesis indicates maladaptation to the unexpected environment of the new era, which increases susceptibility to non-communicable diseases in adulthood (7). These concepts indicate that the upstream risk of metabolic disorders is connected, at least partly, to the maladaptation of developmentally modified phenotypes by evolutionarily acquired plasticity, particularly in response to the expectation of starvation, a strong selection pressure (27, 28).

## Evolutionary Aspect of ‘Metaflammation’; Evolutionary Conservation of Crosstalk Between Immune and Metabolic Pathways

The pathogenesis of obesity with various metabolic disorders is based on a close relationship between nutrient excess and the activation of the innate immune system in the majority of organs involved in energy homeostasis (8, 9, 29–31). Increasing evidence indicates that inflammation occurs with obesity and may play a causative role not only in the development of insulin resistance and disruption of other aspects of energy homeostasis, but also in the augmentation of fat accumulation (9, 29). The characteristics of obesity-associated chronic inflammation differ from other general inflammatory paradigms in that it involves tonic activation of the innate immune system, which has an impact on metabolic homeostasis, generally for a lifetime, and affects multiple organs, such as adipose tissue, the pancreas, liver, muscle, and brain (9, 29). This led to the establishment of the concept of ‘Metaflammation’ (8, 9).

In addition to starvation, infection is a strong selection pressure in animals (32). The avoidance of these two major selection pressures through adjustments to nutrient and immune conditions has been the most important task for animals to survive for hundreds of millions of years (32, 33). The strong relationship between nutrient sensing and immune signaling is rooted in their common evolutionary origins. For example, the hematopoietic system, adipose tissue, and liver are all organized in one functional unit in the fat body of *D. melanogaster* (8). This developmental heritage is responsible for the highly overlapping biological repertoire of these organs, their effects on metabolic and immune cells, and the close relationship between immune and metabolic response systems, which supports the concept of ‘Metaflammation’ from an evolutionary viewpoint (8, 9). The fat body of *Drosophila* is capable of sensing both infectious and metabolic disturbances, and studies on *Drosophila* have provided important insights into highly conserved immuno-metabolic pathways in mammals (8, 9).

Accumulated evidence has also highlighted the crucial role of metabolic reprogramming in macrophage activation not only in immuno-metabolic pathways, but also in the pathophysiological concept of ‘Metaflammation’ (34–36). The infiltration of macrophages and also its associated immune cells into metabolic organs, such as the liver, brain, pancreas, and adipose tissue, is an important factor influencing the maintenance of tissue homeostasis as well as the pathogenesis of metabolic disorders (9). Tissue macrophages function as direct modulators of metabolism, for example, by inducing the polarization of macrophages towards a pro-inflammatory (M1-polarized) phenotype that blocks the effects of insulin (37), to which the contribution of epigenomic alterations (38) and macrophage-secreted products (39) has been demonstrated. It is important to note that many other immune cell types, including dendritic cells, mast cells, eosinophils, and lymphoid cells, may also be involved in metabolic tissue homeostasis and the control of glucose metabolism (9). Therefore, the evolutionary preservation of crosstalk between immune and metabolic pathways is one of the principal concepts of metabolic syndrome.

## ‘Metaflammation’ in the DOHaD Scheme

**Figure 1:**
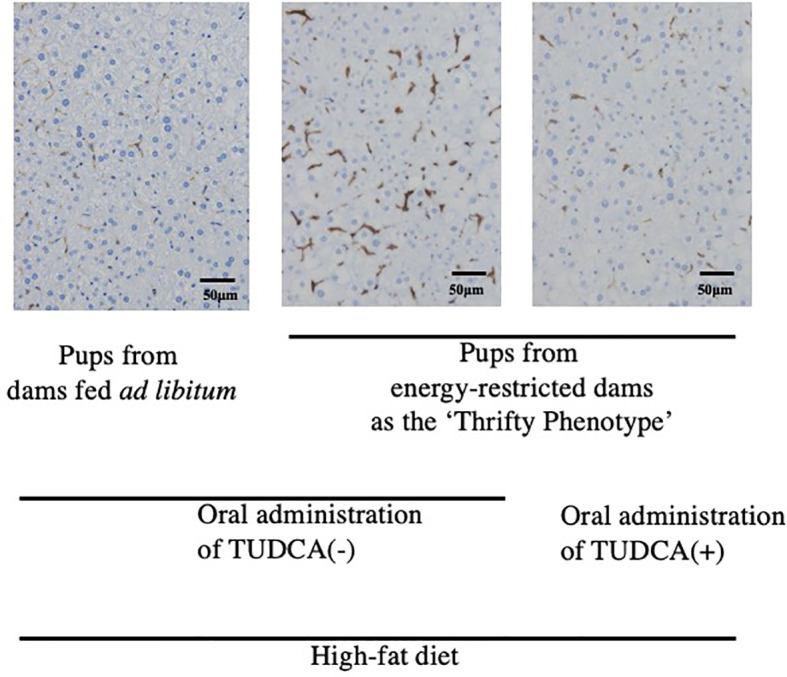
Immunohistochemistry of F4/80-positive hepatic macrophages from 22-week-old pups fed a high-fat diet (Reference 41). Positive staining is brown. TUDCA, Tauroursodeoxycholic acid.

**Figure 2:**
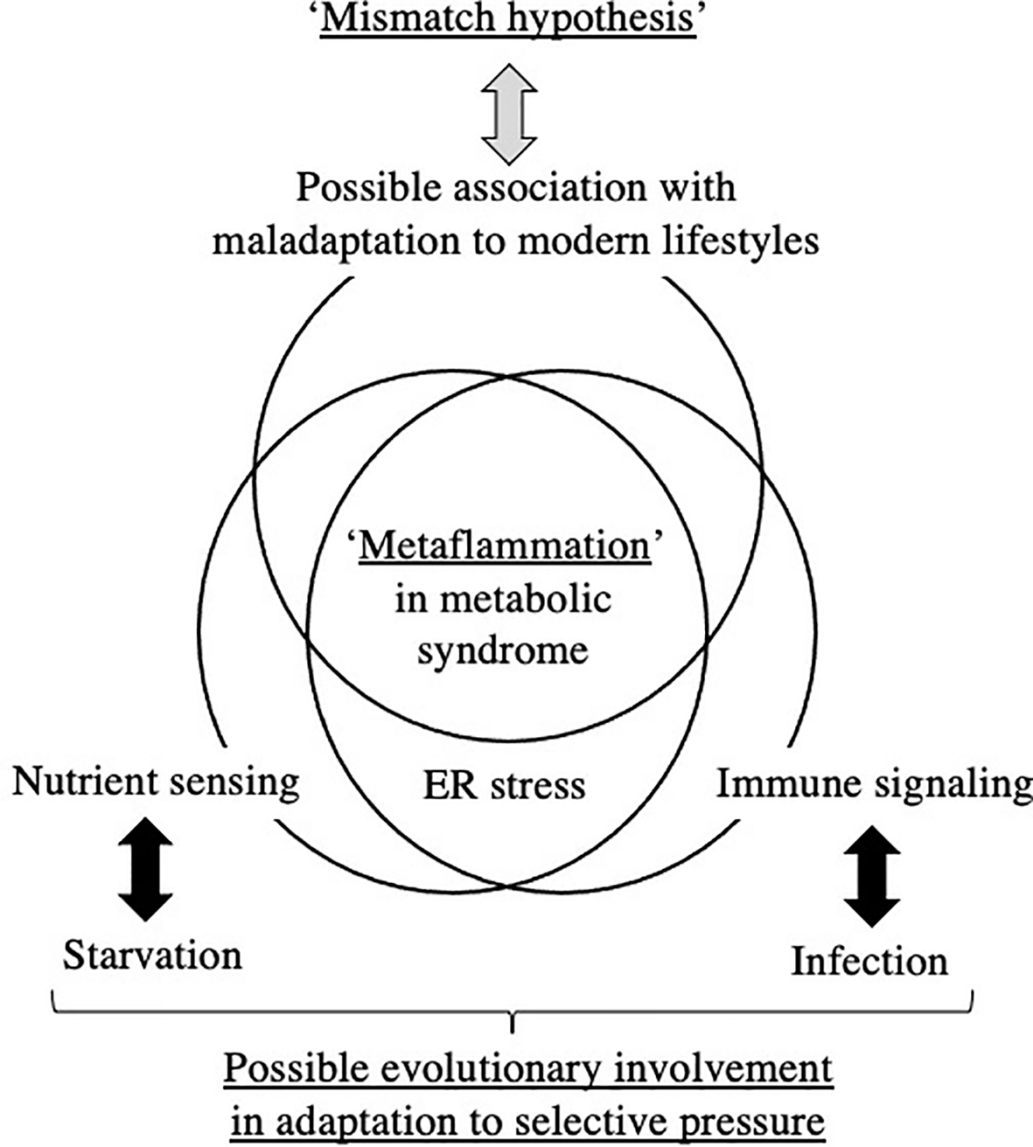
Hypothetical understanding of ‘Metaflammation’ in metabolic syndrome from the viewpoint of the DOHaD theory and evolutionary biology. ER; endoplasmic reticulum.

To the best of our knowledge, limited evidence is currently available to support a direct relationship between ‘Metaflammation’ and the scheme of DOHaD for the pathophysiology of metabolic syndrome. The liver and adipose tissues are representative organs of ‘Metaflammation’, where infiltration of immune cells as well as fat accumulation is frequently observed in metabolic syndrome (8). We developed mice animal model of fetal undernutrition by maternal energy restriction, the offspring of which showed deterioration of fat deposit in the adipose tissue (40) and liver (41) on a high fat diet. Interestingly, the offspring also showed the significant infiltration of macrophages into the adipose tissue (40) and liver (41) (**Figure 1**); thus, we proposed this as a model of ‘Metaflammation’ in the DOHaD scheme. We also demonstrated that intrauterine undernutrition induced significant increases in endoplasmic reticulum (ER) stress markers in the fatty liver of adult pups (41), while the oral administration of the ER stress alleviator, tauroursodeoxycholic acid (TUDCA), markedly ameliorated macrophage infiltration and hepatic steatosis only in pups that experienced undernourishment *in utero* (41, 42) (**Figure 1**). Based on these findings, we propose the involvement of ER stress programming in the developmental origins of ‘Metaflammation’ (**Figure 2**). This speculation is consistent with recent findings showing the critical involvement of ER stress in the co-regulation of chronic inflammation and metabolic disorders (43–45).

Fetal-derived immune cells have been implicated in the development of immune diseases. Mass et al. proposed fetal-derived immune cells as prime transmitters of the long-term consequences of prenatal adversity, namely, inflammatory, degenerative, and metabolic disorders, and, thus, are potential contributors to the DOHaD theory (46). Previous studies suggested the commitment of erythro-myeloid progenitors produced in the extra-embryonic yolk sac to the establishment of long-lasting immunological memory (36, 46–48). Yahara et al. proposed the involvement of erythro-myeloid progenitors in bone regeneration after birth (49). Wu et al. also reported the potential contribution of erythro-myeloid progenitors to homeostasis after birth (50). Nevertheless, the mechanisms by which the memory of tissue-resident macrophages, if actually present, is transferred to mature macrophages remain unclear.

## Discussion

The DOHaD theory is partly derived from retrospective epidemiological observations of susceptibilities to metabolic disorders in offspring that experienced maternal starvation during gestation, such as in the Dutch Famine in World War II (13, 51) and the Great Chinese Famine (52, 53). Since the basic structures of all organs are formed and basic cross-talk between organs is constituted during the embryonic and fetal stages, the ‘Thrifty Phenotype’ hypothesis of acquiring a permanent constitution of low energy consumption in order to adapt to the low nutrient supply *in utero* is plausible (10). The ‘Thrifty Phenotype’ is a type of evolutionarily acquired plasticity in metabolic regulation for humans to survive against the powerful selection pressure of cyclically repeating periods of starvation. However, the ‘Thrifty Phenotype’ mismatches an obesogenic diet and is causatively associated with diabetes, obesity, and associated metabolic disorders in developed and some rapidly developing countries; therefore, the ‘Mismatch’ hypothesis was proposed (7). The essential concepts of the ‘Thrifty Phenotype’ and ‘Mismatch’ hypothesis of the DOHaD theory involve the evolutionary acquisition of plasticity in nutrient sensing against starvation and its maladaptation to an unexpected modern environment (**Figure 2**).

Chronic low-grade inflammation has recently been proposed as a bridge between augmented fat accumulation and metabolic disorders, such as insulin resistance (29); therefore, the concept of ‘Metaflammation’ is now widely accepted (8, 9). The concept of ‘Metaflammation’ is also based on evolutionary adaptation against the selection pressures of starvation and infection, i.e. nutrient sensing and immune signaling (8, 9). The fat body of *Drosophila* is capable of sensing both infectious and metabolic disturbances and evolutionarily differentiated into adipose tissue, the liver, and immune cells in mammals; therefore, this mutual functional control mechanism has been preserved between immune cells and the representative organs of adipose tissue and the liver (8, 9). A similar mutual regulatory mechanism with immune cells has also been proposed in other organs, such as the pancreas and brain, in the concept of ‘Metaflammation’ (9). Therefore, a similar evolutionary trajectory of nutrient sensing and immune signaling underlies the ‘Metaflammation’ concept (**Figure 2**).

The ‘Thrifty Phenotype’ is hypothesized to be advantageous for survival of the fittest in a starved environment; however, to the best of our knowledge, there is limited evidence to support the contribution of ‘Metaflammation’ to survival against infection and/or starvation. The contribution of the potentially long-lasting memory of erythro-myeloid progenitors to the risk of specific diseases in later life, but not to overall survival, has been investigated (36, 46–48). However, we cannot deny the possibility of some unidentified host survival advantage to chronic inflammation or low-grade inflammatory responses incapable of pathogen elimination due to its preservation throughout evolution (**Figure 2**). Although the involvement of crosstalk between immune and metabolic pathways in the acquisition of the ‘Thrifty Phenotype’ in the DOHaD scheme has not yet been elucidated, the evolutionary conservation of this crosstalk has been suggested to contribute to the maintenance of homeostasis in individual organs and is presumably associated with the significant accumulation of fat deposits concomitant with metabolic disruption in metabolic syndrome (**Figure 2**).

Our mouse model revealed that undernourishment *in utero* significantly enhanced the infiltration of macrophages into adipose tissue (40) and the liver (41) (**Figure 1**) only in mice fed a high-fat diet, and this was concurrent with the deterioration of metabolic disorders. We previously reported that this mouse model of undernourishment *in utero* partly represented the ‘Thrifty Phenotype’ due to low levels of diet-induced thermogenesis and a predisposition to obesity (54). The findings of these animal studies strongly suggest that a ‘Mismatch’ to an obesogenic diet in ‘Thrifty Phenotype’ offspring is causatively associated with a malfunction or imbalance in immunometabolic crosstalk, namely, ‘Metaflammation’, particularly under an obesogenic diet (**Figure 2**). Therefore, a more detailed understanding of the fundamental pathophysiology of ‘Metaflammation’ is needed to clarify plasticity in the memory of tissue-resident immune cells, such as macrophages, from the viewpoint of selective advantages and its mismatch to an unexpected new environment, in addition to the principal concept of evolutionarily conserved nutrient and immune sensing systems.

ER is a major site in cells for protein folding and trafficking and ER malfunctions, such as ER stress, promote the unfolded protein response and activate various stress signaling pathways (43, 45). Previous studies proposed roles for ER stress in the common upstream regulators of immune and metabolic functions in ‘Metaflammation’ (43, 45). In our mouse model of the ‘Thrifty Phenotype’, the oral administration of the ER stress alleviator, TUDCA, to pups significantly ameliorated the infiltration of macrophages in the liver only if they experienced undernourishment *in utero* (41) (**Figure 1**). These findings suggest the importance of the regulation of ER stress as a promising research target upstream of developmentally induced ‘Metaflammation’ (**Figure 2**).

On the other hand, functional ‘Trade-off’ for adapting to the environmental disruption is also an important concept of the DOHaD theory (6). It is known that the immune function of hibernating animals is suppressed during the hibernation period when a large amount of fat is stored (55), suggesting a possible presence of a kind of ‘Trade-off’ between fat accumulation and immune activation for the purpose of adapting to the cyclical transitions between hibernation and activity periods. Since coordinate regulation of nutrient and immune functions is a key concept of ‘Metaflammation’, it might be a clue for understanding the pathogenesis of ‘Metaflammation’ from DOHaD theory, to investigate a possible ‘Trade-off’ in ‘Metaflammation’ between nutrient sensing and immune signaling systems in response to the environmental diversity.

In conclusion, in consideration of the ‘Thrifty Phenotype’ and ‘Mismatch’ hypothesis in the DOHaD theory, a promising research target is ‘Metaflammation’ from the viewpoint of selective advantages and its mismatch to an unexpected modern environment, in addition to the principal concept of evolutionarily conserved nutrient sensing and immune signaling systems.

## Author Contributions

All authors listed have made a substantial, direct, and intellectual contribution to the work and approved it for publication.

## Funding

This work was supported by JSPS KAKENHI Grant Numbers JP20H03823, JP20K09666, and JP20K16886, and AMED under Grant Number JP20gm1310009.

## Conflict of Interest

The authors declare that the research was conducted in the absence of any commercial or financial relationships that could be construed as a potential conflict of interest.

## Publisher’s Note

All claims expressed in this article are solely those of the authors and do not necessarily represent those of their affiliated organizations, or those of the publisher, the editors and the reviewers. Any product that may be evaluated in this article, or claim that may be made by its manufacturer, is not guaranteed or endorsed by the publisher.
